# Supplementary material for: Time‑resolved multi-omic analysis of paclitaxel exposure in human iPSC‑derived sensory neurons unveils mechanisms of chemotherapy‑induced peripheral neuropathy
Source: Cell Death Dis. 2026 Feb 10;17(1):211. doi: 10.1038/s41419-026-08445-2 (PMC12921266; doi:10.1038/s41419-026-08445-2)
Supplement: Supplementary file 15 — Dataset 1. [file 41419_2026_8445_MOESM15_ESM.zip › CDD_Open_Data/04_RNA_Seq/Timeline_panel_plots_etc_2023-01-23_1609.zip!/Panel_Plots_etc/Evidence_Plots/72h/evidence_plot_standard contrasts_msigdb_hallmark_72h_PTX_vs_DMSO_ID0_M5924_HALLMARK_MTORC1_SIGNALING.pdf]

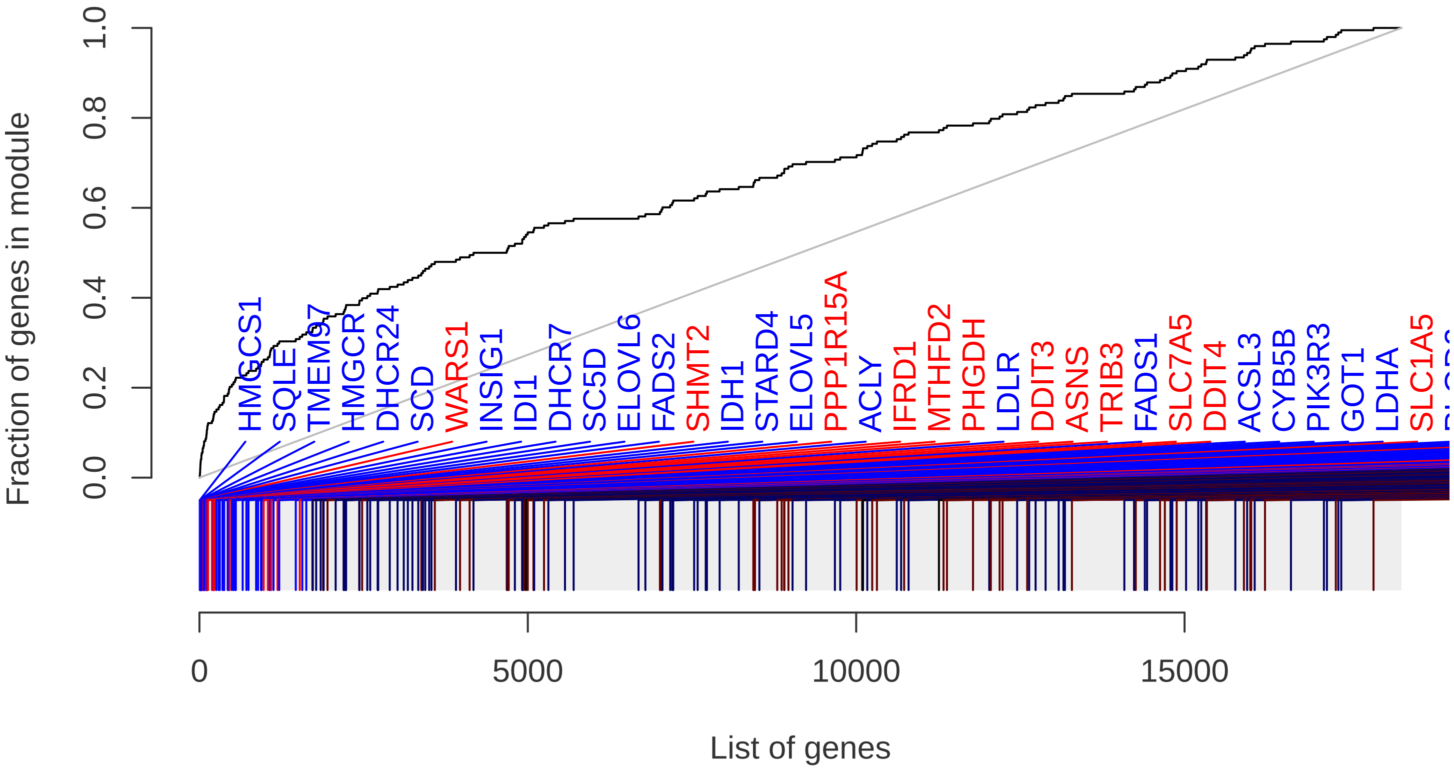
Fraction of genes in module
0.0
0.2
0.4
0.6
0.8
1.0
HMGCS1
SQLE
TMEM97
HMGCR
DHCR24
SCD
WARS1
INSIG1
IDI1
DHCR7
SC5D
ELOVL6
FADS2
SHMT2
IDH1
STARD4
ELOVL5
PPP1R15A
ACLY
IFRD1
MTHFD2
PHGDH
LDLR
DDIT3
ASNS
TRIB3
FADS1
SLC7A5
DDIT4
ACSL3
CYB5B
PIK3R3
GOT1
LDHA
SLC1A5
0
5000
10000
15000
List of genes
